# Predicting Long-Term Mortality after Acute Coronary Syndrome Using Machine Learning Techniques and Hematological Markers

**DOI:** 10.1155/2019/9056402

**Published:** 2019-01-30

**Authors:** Konrad Pieszko, Jarosław Hiczkiewicz, Paweł Budzianowski, Jan Budzianowski, Janusz Rzeźniczak, Karolina Pieszko, Paweł Burchardt

**Affiliations:** ^1^University of Zielona Góra, ul. Licealna 9, 65-417 Zielona Góra, Poland; ^2^Clinical Department of Cardiology, Nowa Sól Multidisciplinary Hospital, ul. Chałubińskiego 7, 67-100, Poland; ^3^Department of Engineering, University of Cambridge, Trumpington St, Cambridge CB2 1PZ, UK; ^4^Department of Cardiology, J. Strus Hospital, ul. Szwajcarska 3, 61-285 Poznań, Poland; ^5^Biology of Lipid Disorders Department, Poznan University of Medical Sciences, ul. Rokietnicka 8, 60-806 Poznań, Poland

## Abstract

**Introduction:**

Hematological indices including red cell distribution width and neutrophil to lymphocyte ratio are proven to be associated with outcomes of acute coronary syndrome. The usefulness of machine learning techniques in predicting mortality after acute coronary syndrome based on such features has not been studied before.

**Objective:**

We aim to create an alternative risk assessment tool, which is based on easily obtainable features, including hematological indices and inflammation markers.

**Patients and Methods:**

We obtained the study data from the electronic medical records of 5053 patients hospitalized with acute coronary syndrome during a 5-year period. The time of follow-up ranged from 12 to 72 months. A machine learning classifier was trained to predict death during hospitalization and within 180 and 365 days from admission. Our method was compared with the Global Registry of Acute Coronary Events (GRACE) Score 2.0 on a test dataset.

**Results:**

For in-hospital mortality, our model achieved a *c*-statistic of 0.89 while the GRACE score 2.0 achieved 0.90. For six-month mortality, the results of our model and the GRACE score on the test set were 0.77 and 0.73, respectively. Red cell distribution width (HR 1.23; 95% CL 1.16-1.30; *P* < 0.001) and neutrophil to lymphocyte ratio (HR 1.08; 95% CL 1.05-1.10; *P* < 0.001) showed independent association with all-cause mortality in multivariable Cox regression.

**Conclusions:**

Hematological markers, such as neutrophil count and red cell distribution width have a strong association with all-cause mortality after acute coronary syndrome. A machine-learned model which uses the abovementioned parameters can provide long-term predictions of accuracy comparable or superior to well-validated risk scores.

## 1. Introduction

The term acute coronary syndrome (ACS) refers to many conditions which include non-ST-segment elevation acute coronary syndrome (NSTE-ACS) and ST-elevation myocardial infarction (STEMI). The common cause of these conditions is inadequate blood flow to the myocardium which can be related to acute cholesterol plaque rupture or erosion and thrombus formation. These conditions have a similar presentation, and the most frequent symptom reported by patients is chest pain, which is one of the most common causes of presentation to the emergency room accounting for up to 6% of emergency department attendances and 27% of medical admissions [1]. Current guidelines emphasize the usefulness of established quantitative risk scores for prognosis estimation [2], which is necessary for the adequate and cost-effective provision of evidence-based therapies.

An increased systemic and local inflammation plays a crucial role in the pathophysiology of ACS. Various hematological indices have been reported to be associated with poorer prognosis or the occurrence of major adverse cardiac events after ACS [3]. These indices include neutrophil to lymphocyte ratio (NLR) [4–6], platelet to lymphocyte ratio (PLR) [7], red cell distribution width (RDW) [8], and mean platelet volume (MPV). These studies brought evidence that such nonspecific markers of the inflammatory response are associated with the GRACE score. [9] Moreover, they can improve its discriminative capabilities [10, 11].

Machine learning (ML) is a field of computer science that uses various computational algorithms to give computer systems the ability to progressively improve performance on a specific task with data, without being explicitly programmed. This term describes a vast spectrum of computational methods, many of which like logistic regression have been used extensively in medical sciences for many years [12]. The most state-of-the-art algorithms are currently subject of intense research and have been recently shown to perform on par with trained ophthalmologists in detecting diabetic retinopathy in eye fundus images [13], classify skin lesion images automatically with dermatologist-level accuracy [14], or detect hip fractures from frontal pelvic X-rays [15].

In our previous research, we successfully used ML techniques to predict in-hospital mortality [16]. In this study, we attempt to develop a new tool for long-term risk assessment following ACS and compare its performance with the GRACE 2.0 model. In contrast to existing risk scores, our tool relies on laboratory tests (including hematological indices) and simple measurements (including blood pressure and heart rate), rather than clinical features. The rationale for such approach is the proven association of inflammatory response with ACS outcomes.

## 2. Methods

We retrospectively examined electronic medical records of patients admitted to a cardiology department between January 2012 and December 2016 to select all patients hospitalized because of an ACS. The analyzed group comprised of patients who had their diagnosis confirmed by a cardiologist according to ESC guidelines [2].

5053 individual patients were qualified (1522 with STEMI, 857 with NSTEMI, and 2674 with unstable angina). We analyzed the descriptions of the electrocardiograms in the patient's medical records to identify patients who had an ST-segment elevation (*n* = 1522) or any ST-segment deviation-elevation or depression (*n* = 4420) according to current guidelines.

We obtained information on all-cause death or survival and on the exact date of death from the national death registry one year after the end of data collection. Patients who had incomplete records or had no blood sample taken during hospitalization were excluded from the study. If a patient was admitted with ACS more than one time in the analyzed period, only the last hospitalization was considered.

All patients were treated according to current guidelines and doctor's therapeutic decisions. Each patient had a venous blood sample taken within 30 minutes from admission. The complete blood count and hematological parameters were analyzed using an automated blood cell counter *CD-RUBY* (*Abbott*, *Lake Bluff*, *Illinois*, *USA*). Biochemical parameters were measured using *COBAS 6000* (*Roche*, *Basel*, *Switzerland*). The results of the laboratory tests as well as the clinical information were obtained retrospectively from the electronic medical record (EMR) system at the time of follow-up. During the period of data collection, both Troponin I and Troponin T were used. Therefore, we expressed troponin elevation as a ratio (actual value divided by the norm).

Statistical analyses were performed using the RStudio Software. The Shapiro-Wilk test was used to test the variables' distribution for normality. Most of the analyzed variables did not have a normal distribution. Median and interquartile ranges were selected as measures of central tendency. The univariable two-tailed Mann-Whitney *U* test was used to compare numerical features. We created a multivariable Cox regression model using variables with statistically significant differences (*P* value <0.05) in univariate analysis. 310 observations were excluded from the analysis because of missing values. We did not use automated stepwise backward elimination. Instead, all variables which were suspected to influence the outcome were entered into the model [17]. The list of variables used in the Cox regression model is presented in Table 1. The proportional hazard assumption was verified using Schoenfeld residuals. To assess the time-varying effects of the selected variables, Aalen's additive model was used. A *P* value <0.05 indicated statistical significance. The results were presented as hazard ratios with 95% confidence intervals (CI).

A probability of death during hospitalization and after 6 and 12 months from admission according to the GRACE 2.0 score was calculated using the model coefficients published on the GRACE project website (https://www.outcomes-umassmed.org/grace/). A Python package was developed to allow for the batch calculation of the GRACE 2.0 death probability based on relevant clinical and laboratory features. As the information about Killip class and creatinine level was available for almost all patients, the full version of the algorithm was used. In 84 cases the missing data did not allow for the calculation of the GRACE probability.  Table 1 presents and compares the variables analyzed in the COX regression model as well as the variables used by the ML model and for the calculation of the GRACE score.

### 2.1. Machine Learning Methods

Model selection, optimization, and fitting were performed using the Python 3.6 and scikit-learn software packages. We used 4969 observations for training and evaluating the ML model. We have excluded 84 observations where variables necessary to calculate the GRACE score were missing, as presented in Table 1. The remaining missing values which did not affect the calculation of the GRACE score were imputed using mean of all observations. The gradient-boosted tree algorithm was implemented using the xgboost [18] software package.

One-fifth of the available data (*n* = 994) was put aside as a test set and not used for training. Observations for the test set were chosen randomly, but in a way that preserved the ratio of positive to negative class (death and survival). The ML classifier was optimized using the training data only (*n* = 3975), using the 5-fold cross-validation. In this process, the training data was divided into 5 parts, and each of these parts was used to train the classifier and to measure its performance. We measured the performance of the GRACE score and our model by calculating the areas under Receiver Operating Characteristic (ROC) curves. The performance measurements during cross-validation were averaged and expressed by mean ± standard deviation. Finally, the performance of both classifiers was compared by calculating the areas under the ROC curves on the test set which was not used for training the ML model at all. This process was repeated in identical fashion for all analyzed endpoints: in-hospital death, 6-month death, and 12-month death.

## 3. Results

**Figure 1:**
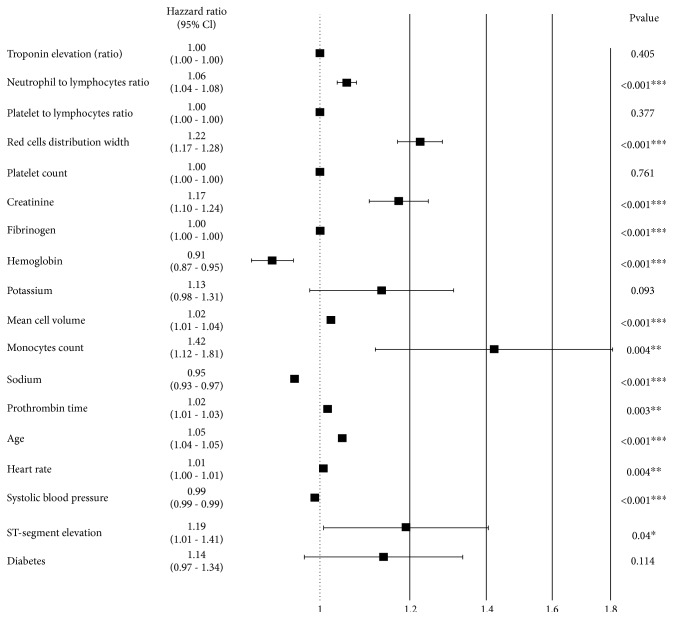
Results of Cox regression. Hazard ratios are presented as black rectangles, and confidence level bands are presented as whiskers. The central vertical line indicates a hazard ratio of 1.

The in-hospital mortality rate was 1.64% (*n* = 83) within 6 months from admission 5.87% (*n* = 297) and within a year from admission 7.85% (*n* = 397). 766 patients (15%) died during the period of the study (from January 2012 until acquisition of the survival data in December 2017). The baseline clinical characteristics and laboratory test results according to survival status are presented in Tables 2 and 3. Some variables including the presence of ST-segment elevation, troponin elevation, sodium levels, and systolic blood pressure did not meet the proportional hazard assumption. However, examining Aalen's additive model indicated that these parameters have a high prognostic value shortly after admission that decreases over time. The results of the multivariable Cox regression analysis are visualized in the form of a forest plot on Figure 1. High RDW, NLR, monocyte count, creatinine level, prothrombin time, age, and heart rate as well as low sodium and hemoglobin were significantly associated with all-cause mortality in the multivariable model. Due to a large number of missing values for CRP and LDL levels, they were not considered for survival analysis, but we kept them in the machine-learned model because of their known association with ACS pathophysiology and outcomes [19].

### 3.1. Machine Learning Results

**Figure 2:**
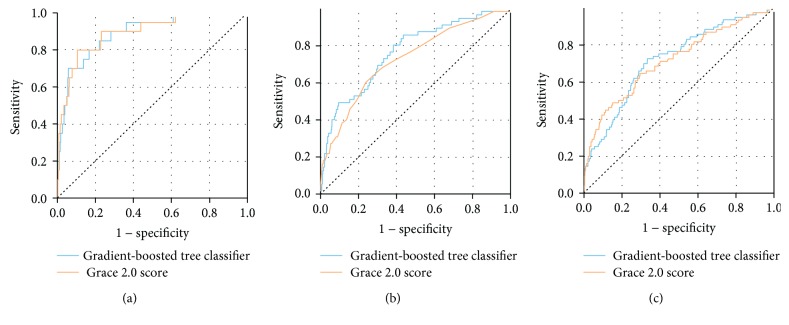
(a), (b), and (c) represent Receiver Operating Characteristic (ROC) curves for in-hospital mortality, 6-month mortality, and one-year mortality, respectively. ROC curves for our classifier are drawn using a blue line, while ROC curves for the Global Registry of Acute Coronary Events (GRACE) 2.0 score are drawn using an orange line.

The model based on the gradient-boosted trees was trained using the following variables as input: troponin elevation ratio, NLR, PLR, RDW, CRP, platelet count, creatinine, hemoglobin, mean cell volume, sodium, prothrombin time, fibrinogen, age, neutrophil count, body mass index, systolic and diastolic blood pressure, heart rate, and sex. The variables were selected to maximize the model's performance, but clinical parameters including the data from the patient's medical history and physical examination were not included in the model. The point was to create a model that could use data that is routinely collected in the EMR system for all patients. The model's performance metrics are summarized in Table 4.  Figure 2 presents the Receiver Operating Characteristic curves for our classifier and the GRACE score 2.0 for the detection of in-hospital, 6-month, and one-year mortality. Eyeballing the Receiver Operating Characteristic (ROC) curves and analysis of areas under these curves (AUROC) reveal that the results of our model and the GRACE score 2.0 are similar. GRACE performed slightly better for short-term results (AUROC 0.9 vs. 0.89) while our model scored better in long-term results (AUROC 0.77 vs. 0.73 and 0.72 vs. 0.71 for 6-month and one-year mortality, respectively).

## 4. Discussion

The results of the survival analysis using Cox regression confirm findings from numerous studies regarding the association of hematological indices including RDW, NLR, and neutrophil count with short- and long-term prognosis after acute coronary syndrome [3]. The low-grade inflammatory process plays an important role in the formation and subsequent destabilization and rupture of the atherosclerotic plaque [20]. In the multivariable Cox regression model, RDW had a strong association with all-cause mortality (HR 1.22, 95% Cl 1.17-1.28). These results are consistent with the findings from other studies that identified RDW as a prognostic marker in cardiovascular diseases and heart failure [21] and also as a predictor of all-cause mortality [22]. It was suggested that patients with increased RDW have lower oxygen supply at tissue level due to decreased red blood cell deformability and impaired blood flow through microcirculation [23]. Our results also seem to confirm the findings from other studies [24] on the impact of admission anemia on long-term prognosis in ACS.

Our model performed better than GRACE score for medium- and long-term prognosis. However, the difference in performance was small, and the calculations of the GRACE scores in our study were made based on retrospective data and could be inaccurate in some cases. This result needs to be confirmed in prospective validation. Better long-term performance of our model might be related to the fact that it uses inflammation biomarkers. The underlying inflammation process is known to be related to atherosclerosis, but the currently used risk scores do not take advantage of this fact.

GRACE score 2.0 has been extensively validated in various populations and proved to have superior discriminatory accuracy for predicting major adverse cardiac events when compared to other risk assessment tools [25, 26]. However, the adoption of its use in a clinical setting was reported to be unsatisfactory. One of the reasons for such situation is the necessity of use of an external application which requires manual data input and consumes extra time [27]. Studies have shown that the integration of risk assessment scores into IT solutions resulted in higher compliance [28]. With all the necessary data available in the electronic medical record system, after integration into existing software, our solution can provide risk assessment without any additional input from the physician. The result could then trigger relevant alerts, helping to select the highest risk patients.

Several studies investigated the application of machine learning techniques to risk stratification in ACS. Most of these studies used data collected retrospectively from a large number of electronic medical reports, similarly as we did in our study [29, 30]. The models they created, however, were based on numerous clinical features, and it is difficult to reproduce the results and apply their solution in a different setting. For instance, VanHouten et al. reported that their machine-learned model could outperform the GRACE score. They used numerous sparse features including the full blood count in most patients and their classifier achieved area under receiver operating curve of 0.85. Our model yields comparable performance, but thanks to using the smaller number of free-of-interpretation features, it is easier to apply and validate externally.

## 5. Study Limitations

In our study, we retrospectively analyzed the electronic medical records of patients hospitalized over several years. This allowed for rapid development on an ML algorithm but is also a significant limitation.

Data stored in medical records are often incomplete, complex, messy, and can be biased [31]. The naive use of raw medical records as input for either inferential statistics or machine learning models can lead to false conclusions. A good example of such situation is the study of Fine et al., in which patients who were admitted with severe community-acquired pneumonia and died in the emergency department had very little information stored in medical records. As a result, some deceased patients appeared healthier than those who survived [32].

The most concerning limitation of our study is related to variables that were stored in medical records as unstructured data in the form of physicians' notes (e.g., descriptions of electrocardiograms). When designing our classifier, we only intended to use features that are available in the medical records as single measurements. Clinical features, including the results of physical examination, patient's symptoms, and medical history, were not considered. This approach is different than those proposed by many other studies exploring the application of machine learning methods in predicting ACS outcomes [29, 30], where all the features that were available in EMR were used. Nevertheless, determining the presence of ST-segment deviation was necessary for calculating the GRACE score. We did not analyze the electrocardiograms directly, and the classification of some ECG descriptions was not obvious. Therefore, the calculations of the GRACE score were especially prone to bias. To make a justified statement on the performance of our classifier vs. any other existing score, it is necessary to evaluate it prospectively, and the scores should be calculated on the day of admission to the hospital.

The follow-up in our study was limited to death or survival status. This is also an important limitation because it was not possible to assess the occurrence of major adverse cardiac events other than all-cause death. Many patients suffered from recurrent ACS, which we did not analyze in this study. Instead, we only took into account the last available hospitalization.

Another important limitation is related to using the Cox regression model. Some of the variables which we used in this model did not meet the proportional hazard assumption. Nevertheless, after analyzing different regression models, we concluded that the predictive value of ST-segment elevation, troponin elevation, sodium levels, and systolic blood pressure may decrease over time and that it is worth presenting the results in this form.

Finally, although the study included patients hospitalized over many years, this dataset is still modest in terms of machine learning model development. The performance of our classifier varied slightly, depending on which observations were chosen randomly for the test set. In contrast, GRACE score was validated on over 100000 patients worldwide, thus the evidence that supports its usefulness is strong. We do not aim to prove that our method is better than any existing well-validated risk score, but to present a new approach to long-term risk prediction in ACS based on different analytic methods and different variables than existing scores.

## 6. Conclusions

Hematological markers of inflammation show strong correlation with the outcomes of ACS, and they can be successfully incorporated into numerical models designed to support clinical decisions. Our model predicted long-term mortality better than GRACE score, but the difference might not be significant, and it requires prospective validation. The potential of such solution lies in taking advantage of the easily available hematological biomarkers and in eliminating the necessity to enter the results of clinical examination or the past medical history into the model.

## Figures and Tables

**Table 1:** Variables used in the Cox regression model, machine-learned model, and for the calculation of the GRACE score.

COX regression	Machine-learned model	GRACE score
*n* = 4743 (310 observations excluded due to missing values)	*n* = 4969 (84 observations excluded due to missing values that where required to calculate GRACE score)	
Troponin elevation ratio	Troponin elevation ratio	Age
Neutrophil to lymphocyte ratio Platelet to lymphocyte ratio	Red cell distribution width	Heart rate
Red cell distribution width	Platelet count	Systolic blood pressure
Platelet count	Creatinine level	Creatinine level
Creatinine level	Hemoglobin level	ST-segment deviation
Fibrinogen level	Mean cell volume	Troponin elevation (true or false)
Hemoglobin level	Sodium level	Killip class
Potassium level	Prothrombin time	
Mean cell volume	Fibrinogen level	
Monocyte count	Age	
Sodium level	Lymphocyte count	
Prothrombin level	Neutrophil count	
Age	LDL level	
Heart rate at admission	CRP level	
Systolic blood pressure	Sex	
ST-segment elevation	Heart rate	
Diabetes	Systolic blood pressure	
	Diastolic blood pressure	
	Body mass index	

LDL: low-density lipoprotein; CRP: C-reactive protein.

**Table 2:** Baseline characteristics according to the survival or death status–numerical variables. Data is presented as median and interquartile range (IQR). *P* values refer to the results of the two-tailed Mann-Whitney *U* test.

	Survival (*n* = 4287)	Death (*n* = 766)	*P* value
Age (years)	65.5 (59.4-73.0)	72.1 (64.4-79.8)	<0.001
Troponin elevation (ratio)	0.9 (0.4-3.9)	3.1 (0.7-38.2)	<0.001
Systolic blood pressure (mmHg)	120.0 (110.0-130.0)	117.0 (110.0-130.0)	<0.001
Diastolic blood pressure (mmHg)	80.0 (75.0-90.0)	78.0 (70.0-85.0)	<0.001
Heart rate at admission	72.0 (64.0-83.0)	76.0 (66.0-89.0)	<0.001
C-reactive protein (mg/dl)	0.4 (0.2-1.8)	2.2 (0.6-6.4)	<0.001
Fibrinogen (mg/dl)	398.0 (344.0-467.0)	430.0 (363.8-517.2)	<0.001
Creatinine (mg/dl)	1.0 (0.8-1.1)	1.1 (0.9-1.4)	<0.001
Neutrophil (10^3^/mm^3^)	5.1 (3.9-6.6)	6.1 (4.5-8.6)	<0.001
Lymphocyte (10^3^/mm^3^)	1.9 (1.5-2.4)	1.6 (1.2-2.2)	<0.001
Monocyte (10^3^/mm^3^)	0.6 (0.5-0.7)	0.7 (0.5-0.9)	<0.001
Eosinophil (10^3^/mm^3^)	0.1 (0.1-0.2)	0.1 (0.0-0.2)	<0.001
Hematocrit (%)	42.7 (39.8-45.4)	40.3 (35.9-43.9)	<0.001
Hemoglobin (g/dl)	14.5 (13.5-15.5)	13.5 (11.9-14.8)	<0.001
Red cell distribution width	12.2 (11.6-12.9)	12.8 (12.0-14.0)	<0.001
Mean cell volume (fl)	91.1 (88.2-94.4)	91.6 (88.1-95.2)	0.1
Platelets (10^3^/mm^3^)	221.0 (186.0-261.0)	223.0 (174.2-269.0)	0.72
Mean platelet volume (fl)	8.6 (7.5-9.8)	8.3 (7.3-9.6)	0.001
Alanine aminotransferase (U/l)	24.0 (17.0-34.0)	23.0 (16.0-37.0)	0.01
Aspartate aminotransferase (U/s)	24.0 (19.0-32.0)	28.0 (20.0-52.0)	<0.001
Basophil count (10^3^/mm^3^)	0.1 (0.1-0.1)	0.1 (0.0-0.1)	0.02
Cholesterol level (mg/dl)	175.0 (144.0-216.0)	162.0 (135.0-198.0)	<0.001
Low-density lipoprotein (mg/dl)	106.0 (78.0-143.0)	97.0 (73.0-127.0)	<0.001
High-density lipoprotein (mg/dl)	50.0 (41.0-60.0)	46.0 (37.0-57.0)	<0.001
Triglycerides (mg/dl)	122.0 (88.0-172.0)	105.0 (79.8-149.2)	0.04
Sodium (mmol/l)	141.0 (139.0-143.0)	140.0 (138.0-142.0)	<0.001
Potassium (mmol/l)	4.4 (4.1-4.7)	4.4 (4.1-4.8)	0.01
Urea (mg/dl)	36.0 (30.0-45.0)	47.0 (36.0-65.2)	0.4
Neutrophil to lymphocyte ratio	2.6 (1.9-3.7)	3.6 (2.4-5.9)	<0.001
Platelet to lymphocyte ratio	114.4 (87.8-149.6)	131.6 (94.4-187.3)	<0.001
Days of hospitalization	4.0 (3.0-5.0)	4.0 (3.0-7.0)	<0.001

**Table 3:** Baseline characteristics according to the survival or death status–categorical variables.

	Survival (*n* = 4287)	Death (*n* = 766)
Sex		
Male	2908	493
Female	1379	273
Diabetes		
No diabetes	3106	503
Type 1 diabetes	20	2
Type 2 diabetes	1161	261
PCI during hospitalization		
True	2565	468
False	1722	298
Killip Class		
I	4196	708
II	74	24
III	9	9
IV	8	25
ST-segment elevation		
False	3047	484
True	1240	282
ST-segment deviation		
False	3521	699
True	766	67

**Table 4:** Performance of classifiers in predicting death during hospitalization, within 6 months, and one year after admission.

	In-hospital mortality	6-month mortality	One-year mortality
Our algorithm on validation set	0.85 ± 0.04	0.78 ± 0.03	0.78 ± 0.03
GRACE 2.0 on validation set	0.89 ± 0.04	0.77 ± 0.03	0.76 ± 0.03
Our algorithm on test set	0.89	0.77	0.72
GRACE 2.0 on test set	0.90	0.73	0.71

## Data Availability

The datasets used and analyzed during the study contain at least four indirect identifiers of patients which were used as input variables for machine learning algorithms (sex, age, weight, height, and place of treatment). For this reason, the data cannot be made publicly available in this form. However, authors are willing to share their data on reasonable request and after case-by-case assessment of such request by a local ethics committee.

## Abbreviations

ACS: Acute coronary syndrome
AUROC: Area under the receiver operating characteristic curve
BMI: Body mass index
CABG: Coronary artery bypass grafting
CRP: C-reactive protein
EMR: Electronic medical records
GFR: Glomerular filtration rate
GRACE: Global registry of acute coronary events
HDL: High-density lipoprotein
HR: Hazard ratio
IQR: Interquartile range
LDL: Low-density lipoprotein
MCV: Mean cell volume
ML: Machine learning
MPV: Mean platelet volume
NLR: Neutrophil to lymphocyte ratio
NSTE-ACS: Non-ST-segment elevation acute coronary syndrome
NSTEMI: Non-ST-segment elevation myocardial infarction
PLR: Platelet to lymphocyte ratio
RDW: Red cell distribution width
ROC: Receiver operating characteristic
STEMI: ST-segment myocardial infarction.
